# Social determinants of health: the role of effective communication in the COVID-19 pandemic in developing countries

**DOI:** 10.1080/16549716.2020.1788263

**Published:** 2020-07-13

**Authors:** Ochega A. Ataguba, John E. Ataguba

**Affiliations:** a Centre for Film and Media Studies, Faculty of Humanities, University of Cape Town, Rondebosch, South Africa; b Health Economics Unit, School of Public Health and Family Medicine, Faculty of Health Sciences, University of Cape Town, Observatory, South Africa

**Keywords:** COVID-19, social determinants of health, effective communication, developing countries

## Abstract

The coronavirus disease 2019 (COVID-19) pandemic has affected many countries with increasing morbidity and mortality. Interestingly, many of the actions and policies adopted in countries are linked to the social determinants of health (SDH). The SDH are critical determinants of health and health inequalities that are not directly within the health sector. Policies such as social distancing, good hygiene, avoiding large gatherings, cancelling of social and sports events, using personal protective equipment, schools and restaurants closure, country lockdown, etc. are not necessarily within the health sector but have been promoted to prevent and attenuate COVID-19 infection rates significantly. The SDH that serve to reduce morbidity will forestall or substantially reduce the pressure on many weak health systems in developing countries that cannot cope with increased hospitalisation and intensive health care. This paper argues that one of the most critical social determinants of health (i.e. effective crisis and risk communication), is crucial in many developing countries, including those with fewer confirmed coronavirus cases. We note that the effectiveness of many of the other SDH in reducing the burden of the COVID-19 pandemic hinges on effective communication, especially crisis and risk communication. Although many countries are adopting different communication strategies during the COVID-19 crisis, effective crisis and risk communication will lead to building trust, credibility, honesty, transparency, and accountability. The peculiarity of many developing countries in terms of regional, cultural, linguistic and ethnic diversity is an essential consideration in ensuring effective crisis and risk communication. Developing countries facing significant poverty and disease burden cannot afford to handle the burgeoning of COVID-19 infections and must take preventive measures seriously. Thus, we submit that there is a need to intensify SDH actions and ensure that no one is left behind when communicating crisis and risk to the population to address the COVID-19 pandemic.

## Background

The coronavirus disease 2019 (COVID-19) significantly changed everyday practices, including global public health. Although the initial outbreak was recorded in a province in China [1], within three months, the virus had spread to almost all countries, and it is implicated in more than 346,000 deaths globally [2]. As at 24 May 2020, South Africa reported the highest number of confirmed cases in Africa (22,583), Brazil (365,213) in South America, India (139,049) in Asia, Spain (282,852) in Europe and the USA (1,686,436) in North America [2]. The global nature of the COVID-19 crisis is a threat, posing as ‘an existential crisis of humanity and society’ [3](p.375). This is driving the significant global public health actions and solidarity [3,4], with substantial financial and human resources already deployed by countries to fight the COVID-19 pandemic. In addition to the suspected and confirmed cases, anxiety, panic and uncertainty also accompany the COVID-19 pandemic [4] and could perpetuate and exacerbate health and social inequalities within and between countries. Developing countries that look towards developed countries for financial support may face dire resource constraints as many developed countries are overstretched with the domestic response to the COVID-19 pandemic in their own countries. Without a doubt, the COVID-19 pandemic will have short-, medium- and long-term impacts on economies, especially those with fragile and weak health systems [4,5] and challenge public health systems and their ability to communicate with their populations effectively [6].

In this paper, we argue that apart from changing how health services are delivered and how health systems respond to crisis, the COVID-19 pandemic highlighted the significance of the social determinants of health (SDH), including crisis and risk communication in reducing disease burden. This is especially the case in developing countries where the number of confirmed cases of COVID-19 infections is slowly rising. We note that the SDH represent one of the most significant approaches, championed by the World Health Organization (WHO) and other leading international and regional organisations such as the centres for disease control and prevention, as part of the collective efforts to address the COVID-19 pandemic. The SDH are essential factors that affect people’s health and inequalities in health but lie outside the health sector. The SDH recognise the importance of synergy between different sectors, especially the social sectors in a locality. They include the living conditions and practices, and a broader set of forces and systems that shape these conditions and practices [7].

## Effective communication is a crucial social determinant of health for the COVID-19 crisis

Most approaches adopted in countries to forestall or reduce the infection rate and the transmission of COVID-19, and to some extent, its management, form parts of the SDH. These approaches are inherently multisectoral and are primarily outside the health sector. They include social distancing, using personal protective equipment like wearing a face mask or hand gloves, avoiding social events or large gatherings, school closure, travel restriction, practising good hygiene such as frequent hand washing or sanitising, staying or working from home, tracing and quarantining suspected cases, self-isolation and complete lockdown in a country or region. Although the SDH discourse is not new in public health, the effectiveness of these approaches to contain COVID-19, as we argue, hinges on *effective* communication, including crisis and risk communication [8], a critical SDH. So, apart from the many health systems driven responses adopted by countries to screen for, test and manage COVID-19 cases, the SDH are contributing significantly to reducing the spread of COVID-19 infections. Cumulatively, the pressure on many health systems will be forestalled or reduced through significant actions on the SDH. Arguably, actions on the SDH have also contributed positively to the reduced economic cost of the COVID-19 pandemic [4]. Although the situation is unfolding, with many developing regions, for example, Africa having relatively fewer confirmed cases of coronavirus infections compared to Europe and North America, we submit that addressing the SDH will continue to avert substantial social and economic costs in developing countries that have poor infrastructure and weak health systems [4]. As was learnt from the Ebola crisis in Africa, for example, the fragility and weakness of health systems in many developing countries [9–11] may aggravate the situation as they may not be able to handle the burgeoning of confirmed cases that require intensive care. This highlights the critical role of the SDH in attenuating potential disasters and pressure on health systems.

We argue that substantial coverage of the other SDH exists but not on *effective communication*, an essential SDH (i.e. for mental and physical health) in the current COVID-19 pandemic. Communicating uncertainty and risks about the COVID-19 pandemic, within and between countries, may well have short- and long-term economic impacts, affect morbidity, mortality, trust and reputation through different pathways [6]. In many developing countries, effective communication should be ‘pro-poor’ and ‘pro-vulnerable’. High rurality, low education attainment and limited access to social amenities characterise many developing countries, including in Africa. This means that crisis and risk communication strategies in many of these countries must take cognisance of already existing inequalities and socio-economic fragilities in countries to be effective.

Communication, especially effective crisis and risk communication, that is essential during pandemics [8], including the COVID-19 pandemic should be prominent in many developing countries to, among other things, reduce panic levels and the number of infections significantly [4]. In Africa, for instance, a task force was established by the Africa Centres for Disease Control and Prevention to, among other things, improve risk communication strategies on the continent [12], but apart from a few countries like Kenya and South Africa, many African countries have poorly developed crisis and risk communication strategies. Elsewhere, public risk communication played a critical role in pre-crisis planning efforts for the West Nile Virus epidemic in New York City between 1999 and 2000 [8,13]. During that epidemic, a detailed communication strategy that uses many media outlets was adopted with the New York City Health Commissioner and the mayor as the primary spokespeople. Although the communication strategy was resource-intensive with a few drawbacks, its relative effectiveness hinged partly on its sensitivity to the diversity of people living in New York, including the languages spoken, in disseminating information [13]. Risk communication was also instrumental to Singapore’s success in handling the SARS outbreak in 2003 [8,14]. The leading risk communication recommendation adopted during the SARS outbreak in Singapore was avoiding over-reassurance. Also, through effective risk communication, Singaporeans developed a sense of community spirit, and compassion and respect for others by discouraging external visitors to Singapore during the height of the SARS outbreak [14].

In the wake of the COVID-19 outbreak in China, the WHO swiftly developed a checklist for risk communication and community engagement (RCCE) readiness for countries’ response [6]. Risk communication is primarily about the effective dissemination of high- or low-hazard information to the at-risk population accurately and timeously. On the other hand, crisis communication presumes the existence of an emergency [8]. The WHO checklist guides countries at different stages of the COVID-19 pandemic (including countries with no COVID-19 case reported but preparing for COVID-19 cases, countries with one or more COVID-19 confirmed cases and countries with ongoing COVID-19 transmission) on how to implement effective RCCE strategies that are essential for health emergency readiness and response, to protect their population [6].

Irrespective of the stage of the pandemic in any country, including the stage with no confirmed case, the WHO checklist for RCCE has six domains for actions: (i) setting up, strengthening and managing risk communication systems, (ii) engendering and strengthening internal and partner coordination to harmonise messages and public communication recognising each partners’ strengths and outreach capacities, (iii) timely and effective public communication using appropriate channels and media that target different populations in countries, including ensuring that health professionals are aware of public concerns and have the required training to provide public health advice, (iv) active community engagement appropriate for different audiences including affected people, health care workers, political leaders and donors. For instance, adapting communication materials to accommodate different literacy levels, culture and relevant languages, (v) addressing uncertainty and perceptions, and managing misinformation, and (vi) continuous capacity assessment and capacity building for RCCE as the situation evolves. For example, ongoing training of different stakeholders such as leaders, responders, and spokespeople on RCCE guidance. This move by the WHO, recognising the asymmetry in the perceptions of risks among affected populations, experts and authorities, was a realisation that RCCE is central for successful responses to health emergencies to prevent, among other things, ‘infodemic’ where an excessive amount of information about the COVID-19 pandemic makes it challenging to identify a solution or an appropriate cause of action in countries [6].

The effectiveness of different crisis and risk communication efforts and strategies will vary, depending on the stage of the crisis or pandemic in each country [6], and could be affected significantly ‘by complex, confusing, inconsistent, or incomplete risk messages; lack of trust in information sources; selective and biased reporting by the media; and psychological factors (heuristics) that affect how risk information is processed’ [13](p.383). In many rural localities in developing countries, communication via social media and ‘word-of-mouth’ plays a significant role in disseminating information [15]. Information shared via these ‘informal’ platforms is usually unverified and inaccurate and could contribute to considerable infodemic which may worsen the situation. In fact, as the COVID-19 crisis unfolds, social media communication significantly burgeoned, providing the fertile ground for communicating unverified information with the potential to harm, *inter alia*, public and population health. In response, social media Apps like WhatsApp is limiting to one, the number of contacts that users may forward messages that have been previously forwarded excessively [16]. In some countries, like South Africa, the government sends out SMSes to registered cellphone users in the country with information on where to seek help and assistance. It also urges people to adhere to many measures put in place, including to stay indoors, avoid religious, social and sports gathering, etc. to forestall or attenuate the impact of the COVID-19 pandemic in the country.

The risks associated with miscommunication during the COVID-19 pandemic are undoubtedly high, especially where trust and credibility, for instance, in authorities and governments are eroded. So, ‘communication process must contain elements of trust, credibility, honesty, transparency, and accountability for the sources of information’ [8] (p.35). Importantly, we know that ‘it is perceptions of risk, not actual risk, that determine how people respond to hazards’ [8] (p. 37). So, as a critical SDH, governments in developing countries must ensure that crisis and risk communications strategies engender trust in authority, dispel false and unverified news and information, and contribute to favourable decisions and actions to improve public and population health during the COVID-19 crisis. Besides, such communication strategies in developing countries must leave no one behind [17], especially the poor and vulnerable populations and should be locally relevant in terms of the language and culture. While there is no one-size-fits-all approach or strategy, an initial step is to understand the predominant communication avenues used by different populations in designing appropriate strategies without just replicating traditional approaches [13]. For example, in rural localities, where village chiefs are prominent and well respected, they could serve as avenues for crisis and risk communication. Also, role models, places of worship or religious leaders could be useful for crisis and risk communication.

The poor and vulnerable within and between developing countries already bear a significant burden of disease (i.e. the social gradient) [18–20] that could be exacerbated by the COVID-19 pandemic. We submit that a realisation of the social gradient is important for crisis and risk communication strategies to convey relevant information to target populations for attenuating the already high disease burden and health inequalities. Moreover, as a guide, and in summary, the appropriate risk communication strategies should engage thoroughly with the following [13]: (i) Who is perceived to be the most trustworthy source for providing the information? (ii) Who is best suited to communicate crisis and risk messages at different times and in different environments? (iii) Which messages are most likely to be effective in different circumstances and settings? (iv) Which messages respect the different values, cultures and belief systems? (v) Which messages raise moral or ethical issues? And ultimately, (vi) where, when, and how to communicate risk information to different population groups.

## Conclusion

Addressing the SDH is essential for reducing health inequalities [7,21–26], especially in developing countries with weak health systems. Thus, we argue that the COVID-19 outbreak that is compounding morbidity and mortality has put a spotlight on the SDH as central for improving population health in developing countries. Developing countries must, therefore, leverage on key SDH to forestall congestion or decongest their already fragile health systems and reduce the potential negative impacts of the COVID-19 pandemic in their countries. In addition to the many preventive measures already in place, developing countries must take advantage of another essential SDH – effective communication, including crisis and risk communication – without leaving anyone behind to address the COVID-19 pandemic. Among other things, the WHO checklist for RCCE [6] is an important starting point for countries to improve effective communication and community engagement.

## Paper context

The COVID-19 pandemic has heightened the critical roles of the social determinants of health for reducing health inequalities. Most significantly, the role of effective communication, especially crisis and risk communication, in developing countries. This paper highlights the centrality of effective crisis and risk communication, a critical social determinant of health, for addressing the COVID-19 pandemic, to forestall or reduce the pressure on the weak health systems in many developing countries.

## References

[1] Wu Z, McGoogan JM. Characteristics of and important lessons from the coronavirus disease 2019 (COVID-19) outbreak in China: summary of a report of 72 314 cases from the Chinese center for disease control and prevention. JAMA. 2020;323:1239–5. 32091533 10.1001/jama.2020.2648

[2] Mamoon N, Rasskin G COVID-19 Pittsburg: Navid Mamoon and Gabriel Rasskin; 2020 [cited 2020 525]. Available from: https://www.covidvisualizer.com/

[3] Fuchs C. Everyday life and everyday communication in coronavirus capitalism. tripleC Commun Capitalism Critique. 2020;18: 375–399.

[4] Ataguba JE. COVID-19 pandemic, a war to be won: understanding its economic implications for Africa. Appl Health Econ Health Policy. 2020;18:325–328. 32249362 10.1007/s40258-020-00580-x PMC7130452

[5] McKibbin W, Fernando R The global macroeconomic impacts of COVID-19: seven scenarios. The Brookings Institution Working Paper. Washington DC: The Brookings Institution; 2020.

[6] World Health Organization. Risk communication and community engagement readiness and response to coronavirus disease (COVID-19). Geneva: World Health Organization; 2020 [cited 2020 523]. Available from https://apps.who.int/iris/rest/bitstreams/1272597/retrieve

[7] Commission on Social Determinants of Health. Closing the gap in a generation: health equity through action on the social determinants of health. Final Report of the Commission on Social Determinants of Health. Geneva: World Health Organization; 2008.

[8] Glik DC. Risk communication for public health emergencies. Annu Rev Public Health. 2007;28:33–54. 17222081 10.1146/annurev.publhealth.28.021406.144123

[9] Kieny M-P, Evans DB, Schmets G, et al. Health-system resilience: reflections on the Ebola crisis in western Africa. Bull World Health Organ. 2014;92:850. 25552765 10.2471/BLT.14.149278 PMC4264399

[10] Kruk ME, Myers M, Varpilah ST, et al. What is a resilient health system? Lessons from Ebola. Lancet. 2015;385:1910–1912. 25987159 10.1016/S0140-6736(15)60755-3

[11] O’Hare B. Weak health systems and Ebola. Lancet Glob Health. 2015;3:e71–e72. 25617195 10.1016/S2214-109X(14)70369-9

[12] Makoni M. Africa prepares for coronavirus. Lancet. 2020;395:483. 32061284 10.1016/S0140-6736(20)30355-X PMC7133753

[13] Covello VT, Peters RG, Wojtecki JG, et al. Risk communication, the West Nile virus epidemic, and bioterrorism: responding to the commnication challenges posed by the intentional or unintentional release of a pathogen in an urban setting. J Urban Health. 2001;78:382–391. 11419589 10.1093/jurban/78.2.382 PMC3456369

[14] Lanard J, Sandman PM Sars communication: what Singapore is doing right. The (Singapore) Straits Times. 2003 5 6.

[15] Einarsdóttir J, Passa A, Gunnlaugsson G. Health education and cholera in rural Guinea-Bissau. Int J Infect Dis. 2001;5:133–138. 11724669 10.1016/s1201-9712(01)90087-6

[16] Hern A WhatsApp to impose new limit on forwarding to fight fake news United Kingdom2020 [cited 2020 4 11]. Available from: https://www.theguardian.com/technology/2020/apr/07/whatsapp-to-impose-new-limit-on-forwarding-to-fight-fake-news

[17] Rudd RE, Comings JP, Hyde JN. Leave no one behind: improving health and risk communication through attention to literacy. J Health Commun. 2003;8:104–115. 14692575 10.1080/713851983

[18] Adler NE, Boyce T, Chesney MA, et al. Socioeconomic status and health: the challenge of the gradient. Am Psychol. 1994;49:15–24. 8122813 10.1037//0003-066x.49.1.15

[19] Deaton A. Policy implications of the gradient of health and wealth. Health Aff (Millwood). 2002;21:13–30. 10.1377/hlthaff.21.2.13 11900153

[20] Umuhoza SM, Ataguba JE. Inequalities in health and health risk factors in the Southern African development community: evidence from world health surveys. Int J Equity Health. 2018;17:1–15. 29703215 10.1186/s12939-018-0762-8 PMC5921793

[21] Ataguba JE, Day C, McIntyre D. Explaining the role of the social determinants of health on health inequality in South Africa. Glob Health Action. 2015;8:28865. 26385543 10.3402/gha.v8.28865 PMC4575416

[22] Blas E, Gilson L, Kelly M, et al. Addressing social determinants of health inequities: what can the state and civil society do? Lancet. 2008;372:1684–1689. 18994667 10.1016/S0140-6736(08)61693-1

[23] Ichoku HE, Mooney G, Ataguba JE. Africanizing the social determinants of health: embedded structural inequalities and current health outcomes in Sub-Saharan Africa. Int J Health Serv. 2013;43:745–759. 24397237 10.2190/HS.43.4.i

[24] Marmot M. Global action on social determinants of health. Bull World Health Organ. 2011;89:702. 22084501 10.2471/BLT.11.094862 PMC3209984

[25] World Health Organization. The economics of social determinants of health and health inequalities: a resource book. Geneva: World Health Organization; 2013.

[26] World Health Organization. Rio political declaration on social determinants of health. World conference on social determinants of health. 19–21 October 2011. Rio de Janeiro: World Health Organization; 2011.

